# Training specificity performing single-joint vs. multi-joint resistance exercises among physically active females: A randomized controlled trial

**DOI:** 10.1371/journal.pone.0233540

**Published:** 2020-05-29

**Authors:** Nicolay Stien, Helene Pedersen, Aril Hagen Ravnøy, Vidar Andersen, Atle Hole Saeterbakken

**Affiliations:** Faculty of Education, Arts and Sports, Western Norway University of Applied Sciences, Bergen, Norway; Universidade Federal de Mato Grosso do Sul, BRAZIL

## Abstract

Resistance-training of the lower limbs can be performed using exercises moving one (single-joint exercises) or several joints (multi-joint exercises). This study compared the effects of training one multi-joint exercise (leg press) or two single-joint exercises (leg extension and kickback) on dynamic and isometric strength and the transferability of dynamic strength between exercises. Fifty-three physically active women were randomized to a multi-joint (MJ) training group (age = 21.95±0.82 years, mass = 64.85±5.76 kg, height = 167.35±2.47 cm; n = 20), single-joint (SJ) training group (age = 22.56±1.66 years, mass = 64.85±5.76 kg, height = 165.94±2.84 cm; n = 18), or a control (CON) group (age = 21.27±0.68 years, mass = 68.43±4.86 kg, height = 168.63±2.84 cm; n = 15). The training groups participated in an 8-week supervised single- or multi-joint lower limb training consisting of 18 sessions. Pre- and post-training, six repetitions maximum (RM) and maximal voluntary isometric contraction in the three exercises were assessed, along with electromyography of the superficial quadriceps muscles. Improvements in all dynamic exercises were greatest after training the specific exercises (ES = 1.26–2.14, *P*<0.001–0.025) and all were greater in the training groups than in the CON group (ES = 1.43–3.31, *P*<0.001–0.021). The SJ group improved 6RM in leg extension and kickback more than leg press (ES = 1.51 and 2.04, respectively, *P*<0.001), whereas the MJ group improved leg press 6RM more than kickback (ES = 1.10, *P* = 0.002). However, leg press and leg extension strength improved similarly in the MJ group (ES = 0.54, *P* = 0.072). All strength and electromyographic measures remained unchanged in the CON group (ES = 0.00–0.44, *P* = 0.412–0.966). Improved dynamic strength in leg press, kickback and leg extension is best attained by training the specific exercises, but both training modalities can improve strength across all exercises.

## Introduction

A resistance training program designed to increase muscle strength should include a meaningful manipulation of training variables such as volume, intensity, rest intervals, exercise selection, contraction type, and velocity [1–5]. Regarding exercise selection, some of the most typical exercises for the lower limbs are single-joint exercises (i.e., kickback, leg flexion, and leg extension) and multi-joint exercises (i.e., back squat and leg press) [6–9]. Multi-joint exercises have traditionally been viewed as more effective than single-joint exercises for increasing maximal strength, muscle activation, metabolic stress and to more closely mimic daily tasks and sports-specific movement patterns [7, 10, 11]. In contrast, single-joint exercises have been suggested as beneficial owing to reduced technical and coordinative demands [12, 13]. Additionally, single-joint exercises may be better suited for targeting specific muscles and correcting imbalances between muscle groups compared to multi-joint exercises [10, 14].

As different exercises and joint angles affect the length and moment arm of muscles and thereby their ability to generate force and velocity, the selection between single- or multi-joint exercises is likely to impact the development of the trained muscles [15]. Few studies have compared the specific training outcomes and transferability of strength between isolated single- and multi-joint training [6, 16–18]. Gentil et al. [17] observed similar increases in elbow flexor strength across the groups after 10 weeks performing either pull-downs (multi-joint) or biceps curls (single-joint). Contrastingly, a number of studies have reported superior strength improvements following multi-joint training compared to single-joint training of the lower limbs [6, 11, 18]. However, these studies had some potential limitations that challenge the comparability of the findings. For example, Augustsson et al. [6] excluded assessment of dynamic strength in the single-joint exercises, Paoli et al. [11] utilized a different number of repetitions for the single- and multi-joint groups, and Goncalves et al. [18] used a contralateral design (exercising one leg using multi-joint training and the other leg using single-joint training).

Furthermore, the previous studies comparing single- and multi-joint training have excluded measurements of neuromuscular activity. Since increased strength is mediated by morphological and neural factors [8, 19], assessing muscle strength and electromyography (EMG) concurrently could help identify which factors influence the specificity and transferability of strength. Examining the changes in both isometric and dynamic strength might also provide a deeper understanding of the muscular changes following the two training modalities. Whereas improved dynamic strength is mediated in part by neural and coordinative adaptations [12, 19–23], isometric strength may depend more on muscular adaptations due to the reduced coordinative and technical demands. Therefore, it could be of interest to investigate whether task-specific strength improvements following dynamic exercises are similar when tested isometrically.

The primary muscle groups trained in the multi-joint exercise leg press are the knee extensors and the hip extensors, both of which can be trained in isolation in single-joint exercises such as leg extensions and kickbacks [24, 25]. Since perceived lack of time is among the most commonly reported barriers to participation in exercise [26, 27], training one multi-joint exercise rather than several single-joint exercises could be favorable for many people. However, relatively little is known about the specific training outcomes following single- or multi-joint training and whether performing one multi-joint exercise or two single-joint exercises is more beneficial for increasing strength [6]. To the authors’ knowledge, no comparable study has identified whether potential differences between single- and multi-joint training can be attributed to specificity of the contraction type or movement pattern. The findings could be of importance for athletes and practitioners when designing training programs. Hence, the aim of this study was to compare the training outcomes following single- and multi-joint lower limb training and to examine the possible transferability of dynamic strength between single- and multi-joint exercises targeting the same prime movers. Since comparable studies [6, 11, 18] reporting superior improvements following multi-joint training have been limited due to the aforementioned reasons it was hypothesized, based on the principle of specificity, that both training groups would increase dynamic and isometric strength in their trained exercise more than the other groups, and that the increased dynamic strength would be greater in their trained exercise than in their non-trained exercises.

## Methods

### Study design

A randomized controlled trial was conducted to examine the within- and between-group effects of single- and multi-joint training on dynamic strength (six repetitions maximum (6RM)), maximal voluntary isometric contraction (MVIC), and electromyography (EMG) of the leg extensors during the 6RM lifts. The training groups performed 18 sessions over an eight-week period consisting of leg presses (multi-joint (MJ) group) or kickbacks and leg extensions (single-joint (SJ) group). All groups agreed to refrain from any other lower limb strength training during the intervention. Participants were tested pre- and post-intervention for 6RM and MVIC in the three exercises. EMG activity of the superficial quadriceps muscles (rectus femoris, vastus lateralis, and vastus medialis) were assessed during the dynamic leg press and leg extension. The order of the exercises was randomized in a counterbalanced order.

### Participants

Sixty physically active females volunteered to participate in the study. Of these, seven did not complete the intervention or post-test due to personal reasons (n = 2) or illness or injuries not related to the intervention (n = 5). Fifty-three physically active females completed the training intervention (see Table 1 for baseline characteristics). After pre-testing, participants were randomly allocated to one of the following groups: single-joint training (SJ group; *n* = 18), multi-joint training (MJ group; *n* = 20), or a control group (CON; *n* = 15).

**Table 1:** Physical characteristics, resistance training (RT) experience, six repetitions maximum (6RM) and isometric force (N) at baseline.

	Control group (n = 15)	Single-joint group(n = 18)	Multi-joint group(n = 20)
Age (years)	21.27 (20.59–21.95)	22.56 (20.90–24.22)	21.95 (21.13–22.77)
Height (cm)	168.63 (168.79–171.47)	165.94 (163.10–168.78)	167.35 (164.88–169.82)
Body mass (kg)	68.43 (63.57–73.29)	64.99 (60.30–69.68)	64.85 (59.09–70.61)
RT experience (years)	0.65 (0.20–1.10)	0.77 (0.26–1.28)	1.06 (0.33–1.79)
Leg press 6RM (kg)	98.33 (85.70–110.97)	111.17 (96.93–125.40)	101.25 (91.20–111.30)
Isometric leg press (N)	778.58 (650.13–907.03)	970.51 (720.60–1220.42)	873.26 (732.97–1013.55)
Kickback 6RM (kg)	32.60 (28.97–36.23)	35.71 (30.99–40.42)	32.95 (29.58–36.32)
Isometric kickback (N)	672.97 (581.19–764.75)	797.86 (679.71–916.02)	751.74 (660.99–842.48)
Leg extension 6RM (kg)	51.14 (46.23–56.06)	57.03 (52.13–61.92)	54.33 (49.64–59.01)
Isometric leg extension (N)	357.42 (264.67–450.17)	553.85 (407.09–700.61)	490.97 (355.16–626.78)

Values are presented as mean (95% confidence interval).

**Fig 1:**
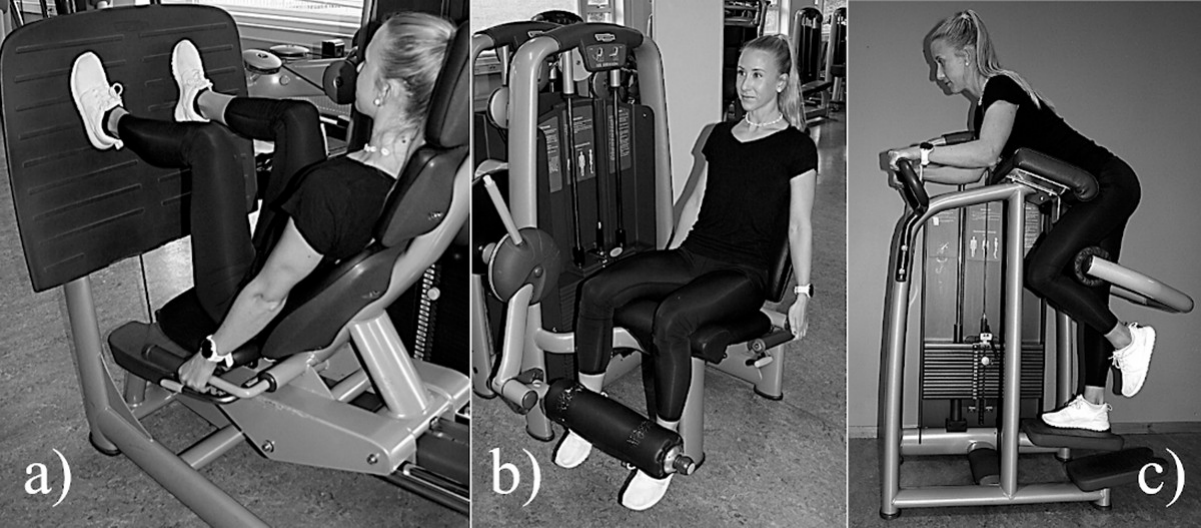
Starting position for the a) leg press, b) leg extension, and c) kickback.

Participants had no injuries and had not performed systematic lower limb strength training (i.e., >1 weekly sessions) in the last 6 months. All participants were informed about the study verbally and in writing and signed an informed consent form before data collection began. The present research procedures conform to the standards of treatment of human participants in research, as outlined in the 5^th^ Declaration of Helsinki, and the preservation of the participants’ privacy was approved by the national committee “Norwegian Centre for Research Data”. The study was also conducted in accordance with the ethical guidelines of Western Norway University of Applied Sciences and Norwegian laws and regulations. The individual depicted in this manuscript (Fig 1) has given written informed consent (as outlined in PLOS consent form) to publish these case details.

### Procedures

All testing and training was conducted between January and March 2019. A familiarization session was conducted 72 hours before the experimental tests. The familiarization session was used to establish the participants’ 6RM and to determine the individual settings for the machines used in training and testing (e.g., seat position and foot placement). The warm-up and strength testing in the familiarization were performed identical as described below for the experimental test. The exercise order was randomized for each participant and was identical in the familiarization session and experimental tests. The intraclass correlation coefficients for the 6RM between familiarization and the experimental session ranged from 0.989 to 0.994, and the coefficient of variation was 1.46%.

Before testing, a light 10-minute warm-up was performed on either a cycle ergometer or on a treadmill, followed by 4 warm-up sets of leg presses with a progressive load based on the participants’ self-reported estimation of 1RM: 1) 20 repetitions at 30%, 2) 12 repetitions at 50%, 3) 6 repetitions at 70%, and 4) 2 repetitions at 80% of 1RM [28]. Three minutes rest were given between each warm-up set and between warm-up and testing [11].

The dynamic leg press, leg extension and kickback were performed in standard commercial training machines (Technogym Selection; Cesena, Italia). A linear encoder (ET-Enc-02, Ergotest Innovation A/S, Porsgrunn, Norway) with a sampling frequency of 100Hz was placed perpendicularly below the weight plates to identify the top and bottom position for each repetition. The encoder was synchronized with EMG measures using the software MuscleLab (v. 8.13, Ergotest Innovation A/S, Porsgrunn, Norway). Although the 6RM was identified during the familiarization session, participants were given up to three attempts in the experimental session to ensure that the correct load was found. A 3–5 minutes rest was given between each 6RM attempt and between each exercise. The repetitions were performed at a controlled, but self-selected tempo without pause between repetitions. When 6 repetitions were successfully completed, the load was increased by 0.5–2.5kg until failure. The 6RM was achieved within one to three attempts. In all dynamic tests, white adhesive tape was placed on the weight magazines indicating where a full extension was reached. The participants were instructed to end the eccentric phase right before the weight plates touched. Verbal encouragement and feedback was given to ensure full range of motion in every repetition.

The 6RM test in the leg press was performed with the feet approximately shoulder-width apart and slightly everted (10–20°). The participants were instructed to keep their knees perpendicularly above the toes and to avoid adduction of the knees. Participants held on to the handles of the machine with their hands to avoid elevating the hip from the seat (Fig 1A). The start position was 90° flexion in the knee joint and 70° in the hip joint, as measured with a goniometer. One complete repetition consisted of extending the knee and hip joints to 180° and 150°, respectively, before lowering themselves back to the start position.

The start position for the dynamic leg extension was 90° flexion in the knee and hip joints. Participants were instructed to grip the handles of the machine to prevent elevation of the hip from the seat. The calf bolster was adjusted to approximately 5 centimeters above the ankle joint (Fig 1B). One repetition consisted of fully extending the knee joint (180°) before returning to the starting position at a controlled pace.

The start position for the kickback was standing with the iliac crest resting on the front bolster of the machine with a 90° angle in the hip joint (Fig 1C). One full repetition was accepted after fully extending the hip joint (180°, leg aligned with the upper body) and bringing the leg back to the starting position without elevating the upper body from the front bolster.

Isometric strength testing was performed in the same machines as used for the dynamic tests. Chains were applied to fixate the machines in the starting position (Fig 1A–1C) while the participants attempted to extend their legs with maximum effort for 5 seconds. A force sensor (Ergotest Innovation A/S, Porsgrunn, Norway) was attached to the chains to register the force output. The results were analyzed with the software MuscleLab (v. 8.13, Ergotest Innovation A/S, Porsgrunn, Norway). Verbal encouragement and visual feedback of the applied force were given to motivate the participants. The maximal voluntary isometric force (MVIC) was calculated from the mean of the 3 seconds with the highest average force output. Two attempts were given with 3 minutes’ rest between the attempts, and the best result was used in the analyses.

To measure EMG activity during the leg press and leg extension exercises, gel-coated electrodes (Dri-stick silver circular sEMG Electrodes AE-131, NeuroDyne Medical, USA; 11mm contact diameter and 2cm center-to-center distance) were used. After careful preparation of the skin in accordance with the recommendations of SENIAM (i.e., shaving, abrasion, and cleaning with alcohol) the electrodes were placed along the presumed muscle fiber direction of the rectus femoris, vastus medialis and vastus lateralis muscles [21]. All electrodes were placed on the dominant leg. The placement of the electrodes relative to anatomical landmarks were as follows: at the half way point from the anterior spina iliaca superior to the superior part of the patella for rectus femoris, four-fifths of the way down the line between the anterior spina iliaca superior and the joint space in front of the anterior border of the medial ligament for vastus medialis, and two-thirds of the way down the line from the anterior spina iliaca superior to the lateral side of the patella for vastus lateralis (www.seniam.org). To reduce noise from the surroundings, the raw EMG signals were amplified and filtered using a preamplifier located close to the sampling point. The preamplifier had a common mode rejection ratio of 100dB and an 8–600Hz sampling frequency. The EMG signals were converted to root mean square (RMS) signals using a hardware circuit network (frequency response 0–600kHz, averaging constant 100ms and total error ±0.5%). The converted RMS signal was sampled at a frequency of 100 Hz using a 16-bit A/D converter. The synchronization of the linear displacement encoder with the RMS data was used to identify the time window for collection of the RMS signal. The stored EMG data was analyzed with the commercial software MuscleLab (v.8.13, Ergotest Innovation A/S, Porsgrunn, Norway). The mean RMS values of the 6 repetitions (from the onset of the first repetition to the end of the last repetition) in the dynamic tests were used for the calculation of the RMS values used in the analyses [29].

### Intervention

The training took place 2–3 times per week on nonconsecutive days over a period of 8 weeks and all sessions were supervised by exercise professionals. All participants attended all the prescribed training sessions. The warm-up intensity and number of warm-up sets were identical as described earlier for the testing sessions. The SJ group trained kickbacks and leg extensions, while the MJ group performed leg presses. Consequently, the SJ group performed twice as many sets as the MJ group, but the sets were divided between the two muscle groups. The progression in sets and repetitions per exercise throughout the intervention is presented in Table 2. Although the kickback machine only allowed unilateral training and testing, participants trained both sides. If all sets within a session were completed with the prescribed number of repetitions and with adequate range of motion and technique (as continuously evaluated by the supervisors), the resistance was increased by 0.5–2.5kg for the next session [7]. The total training volumes for the MJ and SJ groups were calculated as the absolute load lifted throughout the intervention (sets × repetitions × load). This included only the leg press for the MJ group, and the accumulation of leg extension and kickback for the SJ group. Three minutes rest was given between sets. For the kickback, the three minutes started when the first leg was completed.

**Table 2:** Progression in sets and repetitions throughout the intervention period.

Weeks	Weekly sessions	Sets per session	Repetitions per set	Repetitions per session	Repetitions per week
1	2	3	10	30	60
2	2	3	10	30	60
3	2	3	10	30	60
4	2	4	8	32	64
5	2	4	8	32	64
6	3	4	6	24	72
7	3	4	6	24	72
8	2	3	10	30	60

The values represent one exercise per muscle group. The SJ group performed twice the number of repetitions as the MJ group, but divided between the knee extensors and hip extensors.

### Statistical analysis

SPSS version 25.0 (SPSS, Inc., Chicago, Illinois, USA) was used for statistical analyses. Visual inspection of the histograms and the Shapiro-Wilk test were used to examine whether the data was normally distributed. An analysis of covariance (ANCOVA) with Bonferroni post-hoc tests using the pre-test results as covariate was used to analyze the potential differences between and within the groups. Independent samples *t*-tests were used to compare the accumulated training load between the training groups. Statistical significance was accepted at *P*≤0.05. All within-groups results are presented as means with a 95% confidence interval (95%CI) and Cohen’s *d*_*z*_ effect size (ES) as calculated from the change scores divided by their relative standard deviation [30]. The Cohen’s *d*_z_ ES were interpreted as follows: <0.2 = trivial; 0.2–0.6 = small; 0.6–1.2 = moderate; 1.2–2.0 = large; 2.0–4.0 = very large; >4.0 = extremely large [31]. For the between-groups and-exercises differences, Cohen’s *d* ES was calculated as the mean difference between the groups divided by the pooled standard deviation of the change scores. The Cohen’s d ES were interpreted as follows: <0.2 = trivial; 0.2–0.5 = small; 0.5–0.8 = medium; >0.8 = large [32].

## Results

The three groups were homogenous at pre-test for all tested variables (*P* = 0.282–0.635; see Table 1).

**Fig 2:**
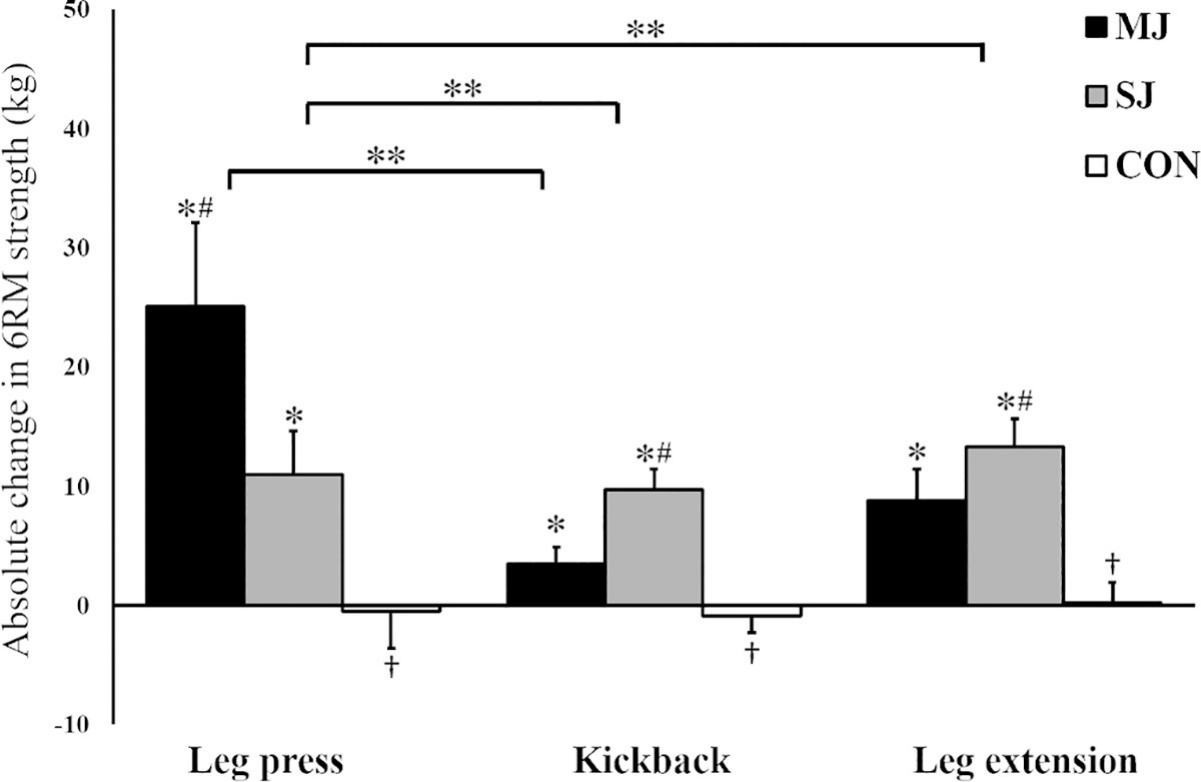
**Absolute change (kg) in 6RM strength from pre- to post-test for the control (white bar), SJ (gray bar) and MJ groups (black bar).** Error bars represent 95% confidence interval. * = Significant change from pre- to post-test (*P* < 0.001). † = Significantly lower change than the training groups (*P* < 0.01). # = Significant difference between training groups (*P* < 0.01). ** = Significant difference between exercises (*P* < 0.01).

The SJ (ES = 1.49–2.75, all *P*<0.001) and MJ groups (ES = 1.05–1.57, all *P*<0.001) increased their 6RM strength in all exercises, whereas the CON group achieved no changes (ES = 0.01–0.22, *P* = 0.412–0.966; Fig 2). There were significant differences between the groups for the change in all exercises (F = 21.367–37.502, all *P*<0.001). In the leg press, the MJ group increased more than the SJ (ES = 1.26, *P*<0.002) and CON groups (ES = 2.21. *P*<0.001), while the SJ group increased more than the CON group (ES = 1.82, *P* = 0.025). In the kickback, the SJ group increased 6RM more than the MJ (ES = 1.64, *P*<0.001) and CON groups (ES = 3.31, *P*<0.001) and the MJ group increased more than the CON group (ES = 1.43, *P* = 0.002). Leg extension 6RM increased more in the SJ and MJ groups compared to the CON group (ES = 2.61, *P*<0.001 and ES = 1.45, *P* = 0.001), whereas the SJ group increased more than the MJ group (ES = 0.51, *P* = 0.017).

When comparing changes in the three exercises within the groups, the MJ group increased leg press 6RM more than kickback (ES = 1.10, *P* = 0.002), whereas no difference was observed between leg press and leg extension (ES = 0.54, *P* = 0.072) or leg extension and kickback (ES = 0.49, *P* = 0.073). The SJ group improved leg extension (ES = 1.51, *P* < 0.001) and kickback (ES = 2.04, *P*<0.001) more than leg press, while no difference was observed between leg extension and kickback (ES = 0.36, *P* = 0.373).

For the MVIC, no between-group differences were observed in any of the exercises (F = 0.562–2.897, *P* = 0.067–0.575).

For the EMG measures, the analyses revealed significant between-group differences for the change in rectus femoris (F = 5.032, *P* = 0.010), but not in the vastus lateralis (F = 2.277, *P* = 0.114) or the vastus medialis (F = 1.300, *P* = 0.283). The SJ group increased rectus femoris EMG (ES = 0.65, *P* = 0.017), and the increase exceeded those observed in the CON group (ES = 1.10, *P* = 0.011), but was not different from the MJ group (ES = 0.26, *P* = 1.000).

Significant differences between the groups were demonstrated for rectus femoris (F = 5.567, *P* = 0.007) and vastus lateralis EMG (F = 3.329, *P* = 0.044), but not for vastus medialis (F = 0.422, *P* = 0.658). Post hoc tests revealed no differences between the groups for vastus lateralis EMG (*P* = 0.053–1.000). The SJ group increased EMG in the rectus femoris (ES = 0.71, *P* = 0.008), and the increase exceeded that of the CON group (ES = 0.77, *P* = 0.007). No other changes from pre- to post-test were observed in any groups (ES = 0.01–0.075, *P* = 0.095–0.847).

**Fig 3:**
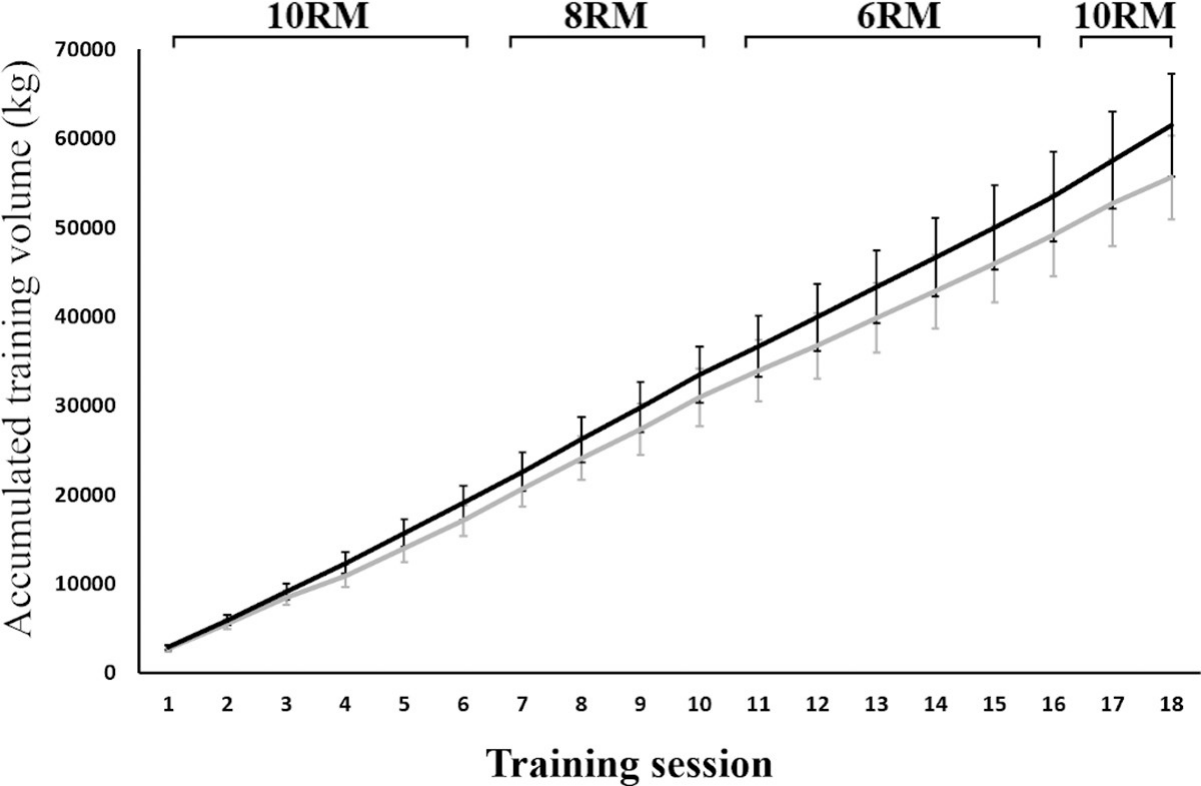
**Accumulated volume (kg) throughout the training period for the SJ (grey line) and MJ group (black line).** Error bars represent 95% confidence interval.

The accumulated training volume was not different between the groups at any point throughout the intervention (4.81–7.71%, *P* = 0.140–0.350; see Fig 3). The total load (kg) lifted after the intervention was 55,610 ± 4,695 kg for the SJ group and 61,456 ± 5,765 kg for the MJ group.

## Discussion

The main findings, as hypothesized, showed that both training groups improved their dynamic strength in all exercises, and the improvements exceeded those observed in the CON group. The improvements in dynamic leg press strength were greater in the MJ group compared to the SJ and CON groups, and the improvements in kickback and leg extension were greater in the SJ group compared to the MJ and CON groups. These results demonstrate task specificity of the movement, suggesting that increased strength in a specific exercise is mediated in part by coordinative improvements specific to the trained exercise.

In line with the hypothesis, all dynamic exercises demonstrated exercise specificity, as both training groups achieved superior improvements in their trained exercises compared to the other groups. Based on the observed improvements for the MJ group, the leg press and leg extension exercises likely offer equally effective training stimuli for the knee extensors because the same range of motion (90°–180° knee angle) is utilized in both exercises. Conversely, analyses of the between-groups differences revealed that training the specific movement still produced the greatest improvements. The greater leg press improvements in the MJ group can be explained by the principle of specificity [33]. Since the leg press is a more complex exercise, the combination of improved muscle strength and movement specific training likely resulted in the superior improvements for the MJ group. More surprisingly, the results for the single-joint exercises contrast with the comparable studies in which multi-joint training has been more effective than single-joint training [6, 11, 18]. These findings could demonstrate movement-specificity despite the low technical demands of the exercise. Furthermore, the leg extensor torque might be different between single- and multi-joint exercises [34], thereby altering the region in the lifts where the resistance is highest. Hence, the observed exercise specificity likely represents adaptations and familiarization to the individual biomechanical demands of the exercises.

The findings for the hip extensors could be related to range of motion [35]. Since the leg press machine kept the participants in a near-supine position with 150° in the hip joint when ending the concentric phase (full hip extension occurs at 180°), the hip extensors were not targeted in their remaining 30° range of motion in this exercise. This was reflected by the lesser improvement observed in kickback strength compared to leg press strength for the MJ group. As we did not measure the EMG activity of the hip extensors, we cannot exclude the possibility that the leg press training also caused a higher activation (i.e., closer to maximal voluntary contraction) of the knee extensors compared to the hip extensors. However, since most of the movement in the leg press occurs around the knee joint, the training stimulus for the hip extensors was probably greater for the SJ group, who targeted the hip extensors to a complete extension in the kickback exercise.

The literature on this subject is not conclusive [6, 11, 17, 36] and owing to variations in methodological approaches, the findings are difficult to compare. To the authors’ knowledge, few studies [11, 18] have included strength testing of both the multi- (back squat or leg press) and single-joint (knee extension) exercises in the same contraction type as used in training. Both studies [11, 18] reported significant 1RM improvements in both exercises for both groups but greater improvements in the MJ group in both their trained and non-trained exercises when compared to the SJ group. In the present study, only the leg press results are in agreement with these findings. Conversely, the SJ group improved kickback strength more than the MJ group, and the improvements in leg extension were not significantly different between the two groups. Importantly, Paoli et al. [11] did not implement any single-joint exercise for the other prime movers (hip extensors) whereas Goncalves et al. [18] implemented leg extension and leg flexion rather than hip extension for the SJ group. Furthermore, Paoli et al. [11] utilized a higher number of repetitions per set and shorter rests for the SJ group compared to the MJ group (12–18RM vs. 6–8RM, respectively, and 1.5–2 min vs. 2.5–3 min, respectively) to equate the volume between the groups. Consequently, the multi-joint training was potentially more favorable toward the 1RM test [10]. In the present study, the rest periods, relative loads, and number of repetitions were identical in both groups. Additionally, the second exercise for the SJ group trained the other prime movers in the multi-joint exercise, which likely produced a more valid comparison of the distinctive training outcomes following the two training modalities.

Since no change in EMG was observed in the MJ group in any exercises, the increase and transferability of dynamic strength may be attributed to other mechanisms. The findings may be surprising, as early phase strength improvements in untrained participants have been closely linked to motor unit recruitment [8, 19]. Other potential explanations include technical improvements or specific adaptations in the discharge characteristics of motor units [37]. Alternatively, neuromuscular changes may be more prominent in muscles that were not measured in the present study (e.g., hip extensors or leg adductors). In accordance with Augustsson et al., [6] the present findings suggested that single-joint exercises may be more effective than multi-joint exercises for increasing neural drive to the prime movers. Still, the few changes observed in EMG activity were only of moderate meaningfulness (ES = 0.65–0.75), and due to the inherent challenges of using pre- to post-test measures of EMG (electrode placement in particular), these results should be interpreted with caution.

Regarding the isometric measures, none of the training groups achieved improvements that exceeded the CON group in any exercise. Despite the very low technical demands of isometric testing, the increased strength following multi-joint training was not transferable to single-joint exercises targeting the trained muscle groups. The lack of strength transferability between exercises in the MVIC test could indicate that task specificity of the contraction type utilized in training is in part responsible for the high transferability of strength between dynamic exercises [12].

### Limitations

The present study had some limitations that should be considered when interpreting the results. As only physically active females without systematic RT experience were recruited, the findings can not necessarily be generalized to other populations. Importantly, we did not control the nutritional habits of the participants. Furthermore, EMG measurements were only taken at the superficial quadriceps muscles, while no measures were taken for the antagonist muscles or the other prime movers of the trained exercises (i.e., hip extensors). It could be argued that one potential limitation was that the leg press and leg extension exercises were performed bilaterally, whereas the kickback machine only allowed one leg to be trained at a time. However, strength gains in bilateral and unilateral exercises for the lower limbs have previously been shown to be mutually transferrable [4]. Furthermore, both groups performed the warm-up in the leg press. However, since the warm-up loads were sub-maximal and sets not were performed to failure, the warm-up likely did not affect the training outcomes. Finally, the MVIC was only tested in the smallest joint angle used in training (90°). It can be speculated that other results might have been found if several joint angles were tested.

### Conclusions

Improved dynamic strength in all tested exercises proved best attainable by training the specific exercises and both single- and multi-joint training produced improvements in dynamic strength in all exercises that exceeded the changes observed in the CON group. Contrastingly, no between-groups differences were observed for isometric strength. These results might indicate specificity of both the exercise and contraction type used in training.

The present study suggested that improved strength in all the tested exercises can be achieved by performing either single- or multi-joint training. Therefore, recreational athletes can base exercise selection on personal preferences and equipment availability. However, if the goal is increased strength in a specific exercise, exercise specificity should be taken into account when designing training programs. Practitioners who engage in sports including primarily dynamic movements across several joints should focus on multi-joint exercises when training for improved athletic performance.

## Supporting information

S1 Dataset With pre- and post- measurements. (XLSX)
